# Limitations of the Boston Carpal Tunnel Questionnaire in Assessing Severity in a Homogeneous Occupational Cohort

**DOI:** 10.3390/life15010132

**Published:** 2025-01-20

**Authors:** Venera Cristina Dinescu, Marius Bica, Ramona Constantina Vasile, Andrei Gresita, Bogdan Catalin, Alexandra Daniela Rotaru-Zavaleanu, Florentin Ananu Vreju, Lorena Sas, Marius Bunescu

**Affiliations:** 1 Department of Health Promotion and Occupational Medicine, University of Medicine and Pharmacy of Craiova, 2 Petru Rares Str., 200349 Craiova, Romania; venera.dinescu@umfcv.ro; 2 Department of Surgery, University of Medicine and Pharmacy of Craiova, 2 Petru Rares Str., 200349 Craiova, Romania; marius.bica@umfcv.ro; 3 Department of Epidemiology, University of Medicine and Pharmacy of Craiova, 2 Petru Rares Str., 200349 Craiova, Romania; alexandra.rotaru@umfcv.ro; 4 Department of Physiology, University of Medicine and Pharmacy of Craiova, 2 Petru Rares Str., 200349 Craiova, Romania; bogdan.catalin@umfcv.ro; 5 Department of Rheumathology, University of Medicine and Pharmacy of Craiova, 2 Petru Rares Str., 200349 Craiova, Romania; florentin.vreju@umfcv.ro; 6 Department of Anatomy, University of Medicine and Pharmacy of Craiova, 2 Petru Rares Str., 200349 Craiova, Romania; lorena.sas@umfcv.ro; 7 Department of Occupational Medicine, University of Medicine and Pharmacy of Craiova, 2 Petru Rares Str., 200349 Craiova, Romania; marius.bunescu@umfcv.ro

**Keywords:** carpal tunnel diagnosis, subjective assessment tools, nerve conduction studies (NCS), occupational health evaluation, hand function impairment, severity stratification challenges

## Abstract

**Background**: Carpal tunnel syndrome (CTS) is a common peripheral neuropathy, often assessed using the Boston Carpal Tunnel Questionnaire (BCTQ). The BCTQ evaluates symptom severity (SSS) and functional status (FSS) but has limitations in stratifying CTS severity, particularly in severe cases. **Objective**: This study aimed to evaluate the utility of the BCTQ in a homogeneous cohort of female workers engaged in repetitive manual tasks, exploring its correlation with objective clinical measures and its performance in detecting CTS severity. **Methods**: A cross-sectional study was conducted on 24 right-hand-dominant female workers with repetitive occupational tasks. CTS diagnosis was confirmed via clinical and electrodiagnostic criteria. Subjects completed the BCTQ, and correlations between BCTQ scores and objective measures such as median nerve cross-sectional area and nerve conduction studies were analyzed. Statistical analyses included comparisons across CTS severity groups and subgroup evaluations based on age and tenure. **Results**: The BCTQ demonstrated moderate correlations with objective measures, with a strong correlation between SSS and FSS scores (r = 0.86, *p* < 0.001). However, the sensitivity of the SSS and FSS was limited, particularly for severe CTS cases. Paradoxically lower scores in severe cases may reflect questionnaire limitations or adaptive responses. Targeted questions addressing pain and sensory symptoms showed better sensitivity (>80%) and may guide clinicians in identifying slight CTS cases. **Conclusions**: While the BCTQ remains a valuable tool for assessing CTS, its limitations necessitate complementary use of objective diagnostic tools, particularly for severe cases. Future refinements, such as tailored scoring systems and integration with clinical measures, could enhance its diagnostic utility and ensure comprehensive assessment of CTS severity.

## 1. Introduction

Carpal tunnel syndrome (CTS) is among the most prevalent peripheral neuropathies, resulting from compression of the median nerve within the carpal tunnel [1]. This condition manifests as pain, numbness, tingling, and, in advanced cases, motor weakness and impaired fine motor skills, significantly diminishing patients’ daily functioning and quality of life [2]. Accurate assessment of CTS severity is essential for determining appropriate treatment strategies and evaluating clinical outcomes, given the condition’s varied presentation and progression [3].

The Boston Carpal Tunnel Questionnaire (BCTQ) is a widely adopted patient-reported outcome measure that assesses CTS-related symptoms and functional status [4]. Comprising the Symptom Severity Scale (SSS) and the Functional Status Scale (FSS), the BCTQ provides a comprehensive view of patients’ perceived discomfort and disability [5]. Its simplicity and patient-centered design have made it a cornerstone in both clinical and research settings [6]. However, its efficacy in accurately stratifying CTS severity has been a subject of ongoing debate. For severe CTS cases, the BCTQ may exhibit certain limitations. For example, affective responses to pain can amplify symptom reporting, resulting in elevated scores. Conversely, adaptive behaviors or diminished sensory perception in chronic cases may paradoxically lower scores. These contrasting mechanisms—exaggerated symptom reporting and adaptive underreporting—necessitate a nuanced interpretation of BCTQ results, particularly in severe or long-standing CTS cases [7].

Moreover, the specificity of the BCTQ is challenged by overlapping symptoms with other conditions, such as distal polyneuropathy and rheumatoid arthritis, emphasizing the need for objective diagnostic confirmation via electromyography (EMG) or nerve conduction studies (NCS) [8,9]. The subjective nature of patient-reported outcomes and external influences, such as occupational exposures, further complicate the interpretation of BCTQ results [10]. While the BCTQ has been applied across diverse populations, its performance in specific cohorts, such as individuals engaged in repetitive manual tasks, remains underexplored [11].

A significant limitation of the BCTQ lies in its potential to misalign with objective measures of severe CTS. Discrepancies between self-reported symptoms and clinical findings highlight critical gaps in its reliability as a standalone tool for severity stratification [12,13]. Occupational cohorts, particularly those involved in repetitive tasks, present unique challenges, as prolonged exposure to repetitive stress may influence both symptom perception and functional adaptation, complicating questionnaire interpretation [14,15].

Although the relationship between occupational exposure and CTS severity is well documented, limited evidence exists on how these factors interact with patient-reported outcomes like the BCTQ. This study sought to evaluate the BCTQ’s performance within a homogeneous cohort of female workers performing repetitive tasks, assessing its ability to capture CTS severity and correlate with objective clinical measures. The use of a controlled cohort minimizes variability from demographic or occupational differences, enabling a focused analysis of the BCTQ’s utility in this context.

Additionally, this investigation explored the correlation between patient-reported outcomes and objective measures such as NCS and ultrasonographic assessments of the median nerve. While the BCTQ provides valuable insights into patients’ perceived disabilities, its ability to reflect underlying physiological changes remains uncertain. Understanding these correlations could inform more integrated assessment protocols that combine subjective and objective data [16]. NCS, regarded as the gold standard for CTS diagnosis, offers precise measures of nerve dysfunction, providing a benchmark for evaluating the BCTQ’s accuracy [17,18]. By comparing BCTQ scores with NCS findings, this study aimed to identify areas of alignment and discrepancy [19].

The study also examined subgroup variations within the cohort, recognizing that age and occupational tenure may influence both CTS risk and symptom perception [20]. Older workers or those with longer tenure in repetitive tasks may exhibit distinct reporting patterns or adaptive behaviors compared to younger counterparts. Analyzing these variations offers insights into how demographic and occupational factors modulate the BCTQ’s performance.

Finally, this study explored potential improvements to the BCTQ scoring system. Tailored scoring models that account for occupational exposures or incorporate targeted questions about pain characteristics, sensory deficits, or functional impairments could enhance the tool’s sensitivity and specificity for occupational CTS cases. Findings from this research aim to refine the clinical management of CTS, contributing to more robust assessment frameworks that improve diagnostic precision and patient care.

## 2. Materials and Methods

This cross-sectional study was conducted in alignment with the Declaration of Helsinki and received approval from the Ethics Committee of the University of Medicine and Pharmacy of Craiova (Approval No. 130/15 April 2024). Informed consent was obtained from all participants prior to their inclusion in the study, ensuring the research adhered to rigorous ethical standards. The study cohort comprised 24 right-hand-dominant female workers employed in an automotive manufacturing factory, all engaged in repetitive manual tasks. These workers were selected based on inclusion criteria, which required female gender, right-hand dominance, occupational exposure to repetitive manual tasks, and confirmation of CTS through clinical and electrodiagnostic evaluations. Participants were excluded if they had pre-existing neurological disorders, systemic conditions such as diabetes or rheumatoid arthritis that could influence CTS diagnosis, a history of prior CTS surgery, or contraindications to electrodiagnostic testing.

The occupational tasks performed by participants were documented in detail to enhance the reproducibility of the study. They involved the handling of automotive seat covering materials, a process requiring coordinated movements using both hands. This job primarily includes repetitive actions such as stretching, aligning, and fixing fabric or leather onto seat frames. Workers frequently engage in precision tasks such as adjusting material edges, applying adhesives, and smoothing surfaces to ensure proper fit and finish. These activities demand continuous wrist and hand motion, often in awkward or constrained positions, which increases the risk of developing carpal tunnel syndrome due to the repetitive and force-intensive nature of the work.

Confounding variables, such as age, work tenure, and genetic predisposition, were carefully accounted for during the analysis phase. Age can be a confounding factor; however, its influence in our cohort is somewhat limited by the relatively narrow age range of the workers (21–50 years, 95% CI: 37.97–42.45). Work tenure, despite being another potential confounder, was relatively short in this cohort, with a mean duration of 32.25 ± 11.04 months (range: 11–42 months, 95% CI: 29.04–35.46). While prior occupational exposure to activities impacting the carpal tunnel could not be definitively confirmed, this factor may nonetheless contribute to variability in outcomes. Another notable confounder could be related to domestic activities, particularly among women, which might also influence the carpal tunnel and lead to the development of CTS. This approach mitigates bias and tries to support the validity of the findings.

Participants completed the BCTQ, which consists of the SSS and the FSS (Supplementary Materials). In parallel, objective measures were collected through nerve conduction studies, which included assessments of median nerve distal motor latency, sensory latency, and amplitude, as well as ultrasonographic evaluation of the median nerve cross-sectional area. The classification of CTS cases into slight, mild, or severe was based on NCS criteria: the minimum motor amplitude was set at 5 mV, the lower limit of sensory amplitude was 15 µV, and latency at the wrist was considered abnormal if it exceeded 4.5 ms. Based on these criteria, CTS cases were classified into slight, mild, or severe categories. Severe CTS was defined by a latency greater than 4.5 ms, with both motor and sensory functions diminished. Mild CTS was characterized by a latency greater than 4.5 ms, with good motor function but altered sensory function. Slight CTS was identified in cases with good latency and motor function but altered sensory function.

The statistical analysis was conducted using MedCalc version 22.021. Correlations between BCTQ scores and objective measures were analyzed using Pearson and Spearman correlation coefficients. The Pearson coefficient was employed to assess linear correlations for interval-scale data, while the Spearman coefficient was used to evaluate monotonic relationships for ordinal data. The Youden Index (J), which is a statistical measure used to summarize the performance of a diagnostic test with a dichotomous outcome, was also used. It captures the effectiveness of a test by balancing sensitivity (true positive rate) and specificity (true negative rate). A Youden Index value of 1 indicates a perfect test, while a value of 0 implies the test has no diagnostic value. This index is particularly useful because it provides a single metric to evaluate the trade-off between sensitivity and specificity. The calculation of the Youden Index in our study facilitated the identification of optimal thresholds, ensuring the best diagnostic performance for the scales used in CTS assessment. A significance threshold of *p* < 0.05 was applied across all analyses to ensure robustness and precision in the interpretation of the findings. This comprehensive methodological approach sought to address the limitations of subjective and objective measures in assessing CTS severity, providing a detailed framework for future studies in similar occupational cohorts.

## 3. Results

The cohort included 24 female subjects engaged in similar workplace activities, specifically manual tasks involving both hands in an automotive manufacturing factory. The mean age of the subjects was 40.21 ± 7.71 years (range: 21–48; 95% CI of the mean: 37.97–42.45; median: 42 years), with an average tenure at their current workplace of 32.25 ± 11.04 months (Table 1).

Selecting the cohort exclusively from women who predominantly use their right hand as the dominant hand allows for an accurate analysis of exposure and response within a homogeneous group, minimizing potential biases such as gender or extreme age variation.

The prevalence of CTS in the studied cohort was 50%, with 24 hands identified as affected. The mean age of subjects with CTS was 41.79 ± 6.49 years compared to 38.63 ± 8.62 years in those without CTS (*p* = 0.157). The average work tenure was 33.75 ± 10.59 months for those with CTS and 30.75 ± 11.51 months for unaffected subjects (*p* = 0.352) (Table 2).

### 3.1. Symptom Severity Scale (SSS)

The SSS score among subjects with CTS was 2.27 ± 0.76, compared to 1.97 ± 0.63 in those without CTS (*p* = 0.15).

There was no difference (*p* = 0.77) among subjects under and over 40 years, but notably, the SSS score was significantly higher among individuals with a workplace tenure exceeding 24 months (2.27 ± 0.79, *p* = 0.034) than in those without CTS (1.82 ± 0.34) (Table 3).

With the exception of question S2 (“How often did hand or wrist pain wake you up during a typical night in the past two weeks?”), where the SSS score was similar (2.21 ± 0.67 in unaffected individuals vs. 2.21 ± 1.22 in those with CTS) but with a higher frequency of “Severe” and “Very Serious” responses among those with CTS, and question S8 (“Do you have tingling sensations in your hand?”), where the mean score was higher in unaffected individuals (2.46 ± 0.72) than in those with CTS (1.96 ± 0.75), likely due to sensory impairment in CTS patients, the scores for other questions were higher in individuals with CTS. These differences ranged between 3% and 28% (Table 4).

**Figure 1:**
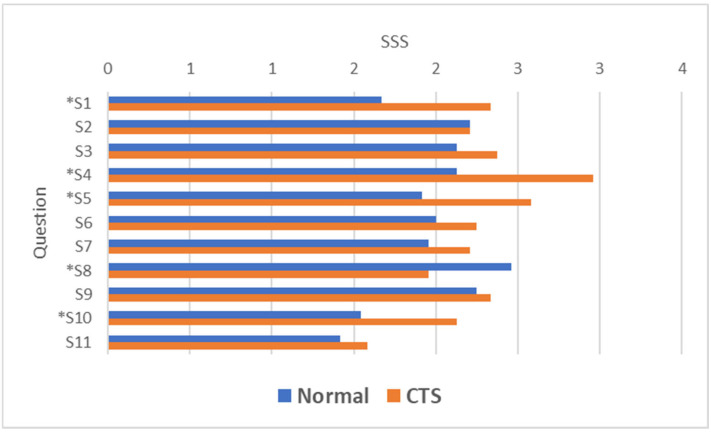
The mean score for the questions in the BCSTQ SSS (* *p* < 0.05—Statistically significant question compared to normal).

Among the nine questions in the BCSTQ questionnaire where SSS values were higher in CTS patients, four questions demonstrated significant differences (*p* < 0.05) (Figure 1).

**Figure 2:**
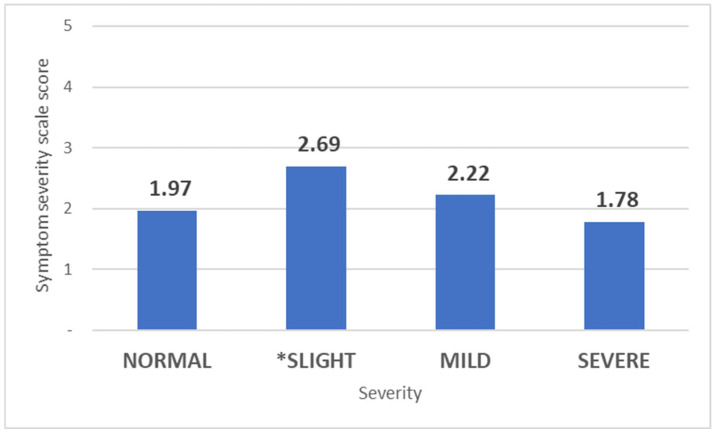
Mean Symptom Severity Scale scores based on CTS severity. The bars represent the mean SSS scores for each category of carpal tunnel syndrome severity: Normal, Slight, Mild, and Severe. (* *p* < 0.05 compared to Normal).

The Symptom Severity Scale of the Boston Carpal Tunnel Questionnaire (11 items) was significantly higher (*p* = 0.025) only in subjects with slight-grade CTS (2.69 ± 0.95) compared to those without CTS (1.97 ± 0.63) when assessing the severity of CTS in affected hands. For individuals with mild CTS, the SSS was 2.22 ± 0.695 (*p* = 0.323), while those with severe CTS paradoxically had a lower SSS score (1.78 ± 0.12, *p* = 0.515) (Figure 2).

**Figure 3:**
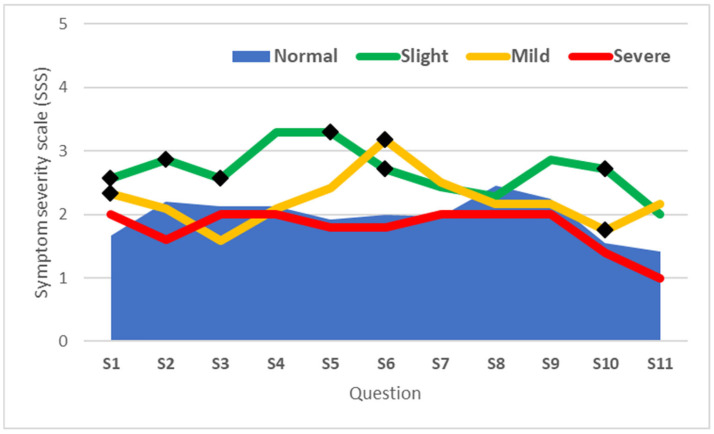
Scores of questions from the BCTQ SSS questionnaire based on CTS severity. The lines represent the mean Symptom Severity Scale (SSS) scores for each question (S1–S11) across categories of carpal tunnel syndrome severity: Normal, Slight, Mild, and Severe. (◆ denotes scores significantly different from the Normal group (*p* < 0.05)).

In cases with slight CTS, 6 out of the 11 questions (S1, S2, S3, S5, S6, and S10) showed statistically significant differences compared to the values recorded for the same questions in the normal group. Conversely, significant differences from the normal group were observed in only 2 out of the 11 questions (S6 and S10) for cases with mild CTS, while no significant differences were noted for cases with severe CTS (Figure 3).

As the Boston Questionnaire is one of the most widely used tools for detecting CTS and is sometimes administered by less experienced medical staff in occupational health settings, its scoring may incorrectly classify CTS cases as normal or less severe. This misclassification risks depriving individuals with the greatest need of timely intervention.

The correlation between the score obtained for each question and the overall SSS score revealed a weak correlation for question S8 (“Do you have tingling sensations in your hand?”) with the SSS score (Spearman’s coefficient rho = 0.14, *p* = 0.34) and a stronger correlation for S4 (How often do you have hand or wrist pain during the daytime?) with a Spearman’s coefficient rho = 0.87 (*p* < 0.001), S6 (Do you have numbness (loss of sensation) in your hand?) with a Spearman’s coefficient rho = 0.85 (*p* < 0.001), and S10 (How often did hand numbness or tingling wake you up during a typical night during the past two weeks?) with a Spearman’s coefficient rho = 0.86 (*p* < 0.001) (Table 5).

No significant correlations were identified between the SSS score and the electromyographic measurements of the median motor nerve at the left or right wrist. However, correlations between the median nerve cross-sectional area and other measured parameters revealed a strong positive correlation with wave amplitude (Pearson r = 0.854; *p* < 0.001) and a small inverse correlation with latency (Pearson r = −0.316; *p* = 0.029) in the right hand (the dominant hand for all subjects). In the left hand, there was a moderate correlation with amplitude (Pearson r = 0.57; *p*< 0.001) and a small correlation with duration (Pearson r = 0.344; *p* = 0.017).

The inability of the SSS to correctly identify severe and mild CTS cases is also suggested by the increase in the AUC amplitude and the Youden index J, from 0.675 and 0.33, respectively, in the analysis of all cases, to AUC = 0.702 and Youden index J = 0.4 in the analysis excluding severe cases and to AUC = 0.732 and Youden index J = 0.46 in the analysis excluding both severe and mild cases.

The sensitivity analysis based on the degree of CTS revealed an increase in sensitivity from 58.33% for all cases to 63.16% when severe cases were excluded and to 71.43% when both severe and mild cases were excluded (Table 6).

The sensitivity of the total SSS score from the BCTQ was determined to be 58.33% at a threshold of >1.82 (95% CI: 36.6–77.9; PLR = 2.33; NLR = 0.56), with an AUC of 0.675 (95% CI: 0.53–0.8; Youden Index J = 0.33; *p* = 0.027). At a threshold exceeding 2, sensitivity decreased significantly to 33.33% (95% CI: 15.6–55.3; PLR = 1.33; NLR = 0.89).

Notably, even when a threshold of >1 was applied (indicating the presence of symptoms), only two questions—S4 (96%) and S7 (91.67%)—achieved sensitivity above 90%. Sensitivity between 80% and 90% was observed for three questions: S1 (83.33%), S6 (83.33%), and S9 (83.33%).

The threshold of >1.82 for sensitivity and specificity analyses was determined through exploratory analysis conducted on both the SSS and the FSS. This approach allowed us to identify a cutoff point that optimally balanced sensitivity and specificity within the context of our cohort. While no direct prior studies specifically supported this threshold, the exploratory analysis was guided by established statistical principles to ensure robustness.

The questions demonstrating sensitivity exceeding 80% primarily assessed critical symptom dimensions: nocturnal pain intensity (S1), frequency of daytime pain (S4), presence of numbness (S6) and tingling (S7), and the nocturnal severity of these symptoms (S9) (Table 7).

The specificity of the SSS score was determined to be 75%. Apart from question S3 (specificity = 95.83%), all other questions demonstrated specificity values below 80%. Questions S1, S5, S10, and S11 exhibited specificity between 50% and 80%, while the remaining questions had specificity values below 50%.

Given that the Boston BCTQ is among the most widely used screening tools, the utility of the SSS score remains limited, particularly in identifying severe and mild cases. Instead, selected questions with reasonable sensitivity may be utilized as valuable individual elements within the assessment framework.

Although the total SSS score did not demonstrate high relevance, targeted questions addressing sensory symptoms such as pain, numbness, and tingling can guide clinicians toward recommending EMG testing.

The use of the SSS score in identifying cases of severe and mild CTS may lead to significant underdiagnosis of actual CTS cases, potentially overlooking critical instances that require attention.

### 3.2. Functional Status Scale (FSS)

The Functional Status Scale score among those with CTS was 1.47 ± 0.54 compared to 1.29 ± 0.49 in those without CTS (*p* = 0.237). The FSS score was significantly higher among individuals with more than 24 months of tenure at their current workplace (1.55 ± 0.55; *p* = 0.001) compared to those with less than 24 months (1.05 ± 0.13) (Table 8).

Of the eight questions included in the Functional Status Scale from the BCTQ, only three showed statistical relevance: F1, which evaluates difficulties with writing; F4, which assesses the difficulty of holding a phone; and F7, which evaluates the difficulty of carrying a full bag. For question F5 (opening jars), the FSS score was unexpectedly higher in unaffected individuals (1.75 ± 1.11; *p* = 0.535) compared to those with CTS (1.56 ± 0.65).

This discrepancy could be attributed to possible adaptation by individuals with CTS, particularly those with longer tenure at their workplace, who may have developed alternative techniques for performing similar tasks. For instance, pronation and unscrewing movements could be strategies adopted to compensate for impaired hand functionality.

**Figure 4:**
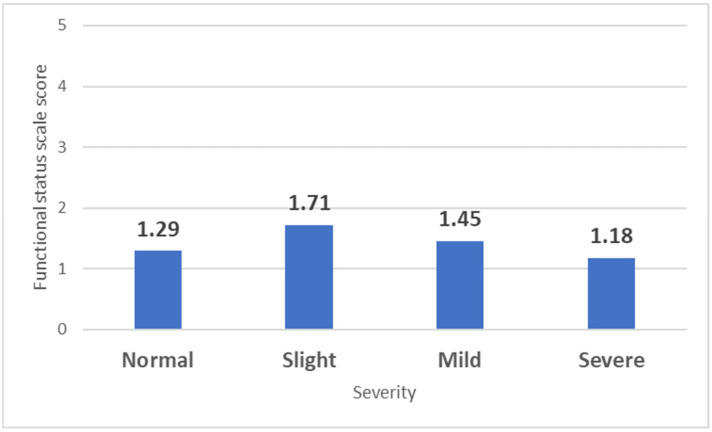
Mean Functional Status Scale (FSS) scores based on CTS severity. The bars represent the mean FSS scores for each category of carpal tunnel syndrome severity: Normal, Slight, Mild, and Severe.

The same pattern observed with the SSS was also noted in the FSS when analyzed based on CTS severity and compared to individuals without CTS (1.29 ± 0.49). A significant difference was observed only in those with slight CTS (1.71 ± 0.67; *p* = 0.049), while those with mild CTS had similar values (1.45 ± 0.51; *p* = 0.378), and individuals with severe CTS even displayed lower scores (1.18 ± 1.19; *p* = 0.61) (Figure 4).

None of the eight questions included in the Functional Status Scale (FSS) recorded a higher score in severe cases than that observed in the normal category.

**Figure 5:**
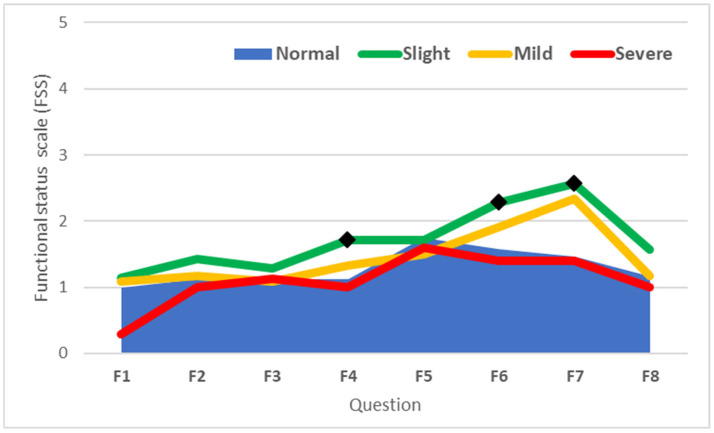
Scores of questions from the BCTQ FSS questionnaire based on CTS severity. The lines represent the mean Functional Status Scale (FSS) scores for each question (F1–F8) across categories of carpal tunnel syndrome severity: Normal, Slight, Mild, and Severe. (◆ denote scores significantly different from the Normal group (*p* < 0.05)).

However, except for F5 (opening of jars), in individuals with slight CTS, the recorded values exceeded those observed in the normal group, and significant differences were recorded for F4 (gripping of a telephone handle, *p* = 0.016), F6 (household chores, *p* = 0.021), and F7 (carrying of grocery basket, *p* = 0.022) (Figure 5).

Similarly, in the case of the FSS score, an improvement in the ability to detect CTS cases was observed among patients with slight CTS. The AUC increased from 0.59 (Youden Index J = 0.25) to 0.61 (Youden Index J = 0.35) when severe cases were excluded from the analysis, and further to 0.66 (Youden Index J = 0.45) when both severe and mild CTS cases were excluded. (Table 9).

At a threshold of >1.38, the sensitivity of the FSS was 37.5% (Youden Index J = 0.25). This sensitivity increased to 47.37% with the exclusion of severe cases and to 57.14% with the exclusion of both severe and mild CTS cases, confirming the improved ability of the FSS score to identify cases of slight CTS (Table 9).

The sensitivity values of the FSS for responses greater than 1 were below 60% for all questions within the FSS score. Sensitivity values of 50% or higher were observed only for questions F6 (58.33%), F5 (50%), and F7 (50%). Accuracy was below 60% for most questions in the FSS score, with F7 showing the highest accuracy at 62.5% (Table 10).

**Figure 6:**
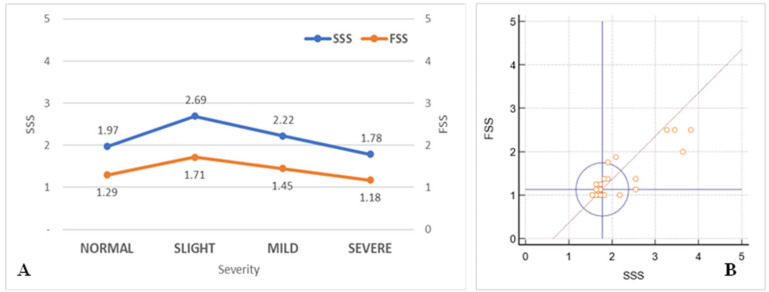
Relationship between SSS and FSS scores. (**A**): Mean Symptom Severity Scale and Functional Status Scale scores across carpal tunnel syndrome severity levels (Normal, Slight, Mild, and Severe). The left y-axis represents the SSS scores, and the right y-axis represents the FSS scores. (**B**): Correlation between SSS and FSS scores. The x-axis represents the SSS scores, and the y-axis represents the FSS scores. The blue circle indicates the 95% coverage probability, while the red line represents the linear correlation.

The correlation between the mean SSS and FSS scores was strong, with r = 0.86 (95% CI: 0.77–0.92; *p* < 0.001), indicating a robust relationship between the responses provided in the two subsets of questions included in the BCSTQ (Figure 6A,B).

## 4. Discussion

The BCTQ is an established tool for assessing CTS. This study provides critical insights into its utility and limitations, particularly within a homogenous cohort of female workers engaged in repetitive manual tasks. The SSS of the BCTQ demonstrated significant promise in detecting slight CTS cases, with specific questions targeting nocturnal pain, daytime pain, and tingling sensations exhibiting high sensitivity. This highlights the potential of the BCTQ as an early screening tool, especially in occupational settings where workers face an elevated risk of cumulative trauma disorders. However, limitations were observed in its ability to stratify severe CTS cases, as paradoxically lower SSS and FSS scores were noted. These findings may reflect sensory adaptation in chronic cases, where prolonged nerve compression results in symptom attenuation. Alternatively, compensatory behaviors such as modifying task execution to accommodate impairments, as well as psychological factors or occupational pressures, may bias self-reported outcomes, diminishing the reliability of the BCTQ in assessing severe CTS.

The FSS, while effective in identifying slight functional impairments, exhibited a ceiling effect that limited its capacity to differentiate more severe functional disabilities. Occupational factors, including job tenure and repetitive task exposure, were associated with higher symptom severity, reinforcing the importance of early workplace interventions such as ergonomic adjustments, job rotation, and task redesign. However, significant differences in symptom patterns between subgroups (e.g., by age or tenure) were not observed, which may be due to the study’s small sample size—a limitation that should be addressed in future research with a larger and more diverse cohort.

Recent studies suggest that integrating patient-reported outcomes like the BCTQ with objective diagnostic tools such as ultrasonography or EMG enhances the accuracy of CTS assessment [21]. Moderate correlations between BCTQ scores and objective measures, such as nerve conduction studies, observed in this study further underscore the need for a multidimensional diagnostic approach. The subjective nature of the BCTQ introduces variability influenced by cultural, psychological, and occupational factors, underscoring its complementary role rather than standalone utility.

To address these limitations, refining the BCTQ with tailored scoring systems or integrating it with novel diagnostics like ultrasound imaging could enhance its diagnostic accuracy and utility across diverse settings. Additionally, the exclusion of male participants, the small sample size, and the lack of longitudinal data to confirm case progression represent study limitations. Future research should address these issues to strengthen the generalizability of findings and explore subgroup differences more comprehensively.

From a practical perspective, this study emphasizes the importance of combining patient-reported outcomes with objective evaluations in workplace settings. For occupational health professionals, integrating tools like the BCTQ with objective diagnostics could facilitate early detection and management of CTS, ultimately improving worker health and productivity. These findings highlight the need for cautious interpretation of BCTQ results and a multidimensional approach to CTS assessment, optimizing both clinical and occupational management strategies.

## 5. Conclusions

The BCTQ remains a valuable tool for assessing CTS, but its limitations in stratifying severity, particularly in severe cases, underscore the need for a multidimensional diagnostic approach. Integrating objective tools, such as electrodiagnostic studies, can enable more comprehensive and accurate evaluations of CTS. This study highlights discrepancies between subjective patient-reported outcomes and objective diagnostic measures, emphasizing the importance of combining these tools for optimal diagnosis and management.

For occupational health professionals, combining the BCTQ with objective assessments in workplace screenings ensures a more comprehensive evaluation. Early interventions, such as ergonomic adjustments and job rotation, should be prioritized to reduce CTS risk among high-exposure occupational groups.

The discrepancies observed in BCTQ results between mild and severe CTS cases may be attributed to factors such as sensory adaptation, compensatory strategies, psychological influences, and occupational pressures. Future research should involve larger, more diverse cohorts and incorporate longitudinal analyses to investigate these dynamics further. Combining objective diagnostic tools, such as ultrasonography or EMG, with the BCTQ could provide deeper insights into the relationship between symptom severity and functional outcomes across different CTS stages.

## Figures and Tables

**Table 1:** Basic characteristics of study group.

Age	% (N)	CTS Grade	% (N)
<40 y	29.2% (14)	Normal	50% (24)
>40 y	70.8% (34)	Slight	14.6% (7)
Work seniority		Mild	25% (12)
<24 m	33.3% (16)	Severe	10.4% (5)
>24 m	66.7% (32)	Total	100% (48)

%(N) represents the percentage of participants within the respective category, with the corresponding number of participants in parentheses, y = years, m = months.

**Table 2:** Median age (years) and work seniority (month) in cases with and without CTS.

	Age (y) M ± DS	*p* *	Work Seniority (m) M ± DS	*p* *
All	40.21 ± 7.71		32.25 ± 11.04	
CTS	41.79 ± 6.49	0.157	33.75 ± 10.59	0.352
Normal	38.63 ± 8.62		30.75 ± 11.51	
Slight	41.1 ± 4.7	0.138	36 ± 8.5	0.278
Mild	43 ± 7.3	0.47	33.5 ± 11.3	0.509
Severe	39.8 ± 7.2	0.774	31.2 ± 13.0	0.945

* compared to normal; y—years; m—months.

**Table 3:** Median score of SSS from BCTQ.

Age		Work Seniority	
<40 y	2.07 ± 0.63	<24 m	1.82 ± 0.34
>40 y	2.014 ± 0.74	>24 m *	2.27 ± 0.79
	*p* = 0.757		*p* = 0.034

* *p* < 0.05, y = years, m = months.

**Table 4:** Comparison of mean symptom severity scores and individual question responses between normal and CTS groups.

	Normal		CTS		
	Mean ± SD	95% CI	Mean	95% CI	*p*
SSS	1.97 ± 0.63	1.71–2.23	2.27 ± 0.76	1.96–2.59	0.15
S1	1.67 ± 0.76	1.345–1.988	2.33 ± 0.96	1.927–2.740	0.011 *
S2	2.21 ± 0.66	1.930–2.486	2.21 ± 1.22	1.695–2.721	1
S3	2.13 ± 0.45	1.936–2.314	2.38 ± 0.71	2.075–2.675	0.152
S4	2.13 ± 0.85	1.766–2.484	2.96 ± 1.16	2.468–3.448	0.008 *
S5	1.92 ± 0.83	1.566–2.267	2.58 ± 1.18	2.087–3.080	<0.001 *
S6	2.00 ± 0.88	1.626–2.374	2.25 ± 0.99	1.832–2.668	0.360
S7	1.96 ± 0.81	1.618–2.299	2.21 ± 0.72	1.904–2.513	0.263
S8	2.46 ± 0.72	2.154–2.763	1.96 ± 0.75	1.641–2.275	0.023 *
S9	2.25 ± 0.79	1.915–2.585	2.33 ± 1.09	1.873–2.794	0.772
S10	1.54 ± 0.83	1.190–1.893	2.13 ± 1.15	1.638–2.612	0.044 *
S11	1.42 ± 0.83	1.066–1.767	1.58 ± 0.83	1.233–1.934	0.4661

* *p* < 0.05, CI = confidence interval, SD = standard deviation.

**Table 5:** Correlation coefficient (Spearman) for individual questions of the SSS with overall symptom severity scores.

	S1	S2	S3	S4	S5	S6	S7	S8	S9	S10	S11
Spearman Coefficient	0.73	0.59	0.71	0.87	0.66	0.85	0.57	0.14	0.53	0.86	0.75
	<0.001	<0.001	<0.001	<0.001	<0.001	<0.001	<0.001	=0.336	=0.001	<0.001	<0.001

**Table 6:** Performance metrics of the Symptom Severity Scale across all cases, excluding severe cases and both severe and mild cases.

	All	Without Severe Cases	Without Severe and Mild Cases
AUC	0.675	0.702	0.732
SE	0.0791	0.0829	0.114
95% CI	0.520 to 0.830	0.539 to 0.864	0.490 to 0.974
*p* (Area = 0.5)	0.0266	0.0149	0.0402
Youden index J	0.3333	0.4035	0.4643
Associated criterion	>1.82	>1.82	>1.82
Sensitivity	58.33	63.16	71.43
Specificity	75	75	75

AUC = Area under the curve, SE= standard error, CI = confidence interval, *p* = significance level.

**Table 7:** Sensitivity and specificity of the total SSS score and its individual questions at a threshold > 1.

	S1	S2	S3	S4	S5	S6	S7	S8	S9	S10	S11
Sensitivity	83.33%	66.67%	4.17%	96%	8.33%	83.33%	91.67%	75%	83.33%	70.83%	41.67%
Specificity	50%	8.33%	95.83%	20.83%	75%	25%	25%	0%	8.33%	62.5%	75%
AUC	0.667	0.375	0.5	0.584	0.417	0.542	0.583	0.375	0.458	0.667	0.583
PLR	1.667	0.727	1	1.213	0.333	1.111	1.222	0.75	0.909	1.889	1.667
NLR	0.333	4	1	0.192	1.222	0.667	0.333		2	0.467	0.778
PPV	62.5%	42.11%	50%	55.81%	25.00%	52.63%	55%	42.86%	47.62%	65.39%	62.5%
NPV	75%	20%	50%	83.33%	45%	60%	75%	0%	33.33%	68.18%	56.25%
Accuracy	66.67%	37.5%	50%	59.18%	41.67%	54.17%	58.33%	37.5%	45.83%	66.67%	58.33%

AUC = Area under the ROC curve; PLR = positive likelihood ratio; NLR = negative likelihood ratio; PPV = positive predictive value; NPV = negative predictive value.

**Table 8:** Functional Status Scale (FSS) scores stratified by age and workplace tenure.

Age		Work Seniority	
<40 y	1.27 ± 0.53	<24 m	1.05 ± 0.13
>40 y	1.43 ± 0.51	>24 m *	1.55 ± 0.55
	*p* = 0.338		*p* = 0.001

* *p* < 0.05 for comparison between groups, y = years, m = months.

**Table 9:** Diagnostic performance of the Functional Status Scale (FSS) across different CTS case exclusions.

	All	Without Severe Cases	Without Severe and Mild Cases
AUC	0.589	0.606	0.658
SE	0.084	0.09	0.129
95% CI	0.437–0.729	0.429–0.784	0.405–0.91
*p* (Area = 0.5)	0.291	0.24	0.221
Youden index J	0.25	0.349	0.446
Associated criterion	>1.38	>1.38	>1.38
Sensitivity	37.5	47.37	57.14
Specificity	87.5	87.5	87.5

AUC = Area under the ROC curve; PLR = positive likelihood ratio; NLR = negative likelihood ratio; PPV = positive predictive value; NPV = negative predictive value.

**Table 10:** Sensitivity and specificity of the FSS score and its component questions at a threshold greater than 1.

	F1	F2	F3	F4	F5	F6	F7	F8
Sensitivity	8.33%	20.83%	12.50%	20.83%	50%	58.33%	50%	20.83%
Specificity	100%	87.5%	87.5%	87.5%	50%	58.33%	75%	87.5%
AUC	0.542	0.542	0.5	0.542	0.5	0.583	0.625	0.542
PLR	-	1.667	1	1.667	1	1.4	2	1.667
NLR	0.917	0.905	1	0.905	1	0.714	0.667	0.905
PPV	100%	62.50%	50%	62.50%	50%	58.33%	66.67%	62.50%
NPV	52.17%	52.50%	50%	52.50%	50%	58.33%	60.00%	52.50%
Accuracy	54.17%	54.17%	50%	54.17%	50%	58.33%	62.50%	54.17%

AUC = Area under the ROC curve; PLR = positive likelihood ratio; NLR = negative likelihood ratio; PPV = positive predictive value; NPV = negative predictive value.

## Data Availability

The data presented in this study are available on request from the corresponding author as the dataset is part of an ongoing research project and will be used for future publications.

## Supplementary Materials

The following supporting information can be downloaded at https://www.mdpi.com/article/10.3390/life15010132/s1.
