# Supplementary material for: Limitations of the Boston Carpal Tunnel Questionnaire in Assessing Severity in a Homogeneous Occupational Cohort
Source: Life (Basel). 2025 Jan 20;15(1):132. doi: 10.3390/life15010132 (PMC11766517; doi:10.3390/life15010132)
Supplement: Supplementary file 1 [file life-15-00132-s001.zip › life-3412355-supplementary.pdf]

Appendix: Boston Carpal Tunnel Questionnaire (BCTQ)

This appendix provides the full details of the Boston Carpal Tunnel Questionnaire (BCTQ), including its two primary components: the Symptom Severity Scale (SSS) and the Functional Status Scale (FSS). These scales are used to assess the severity of carpal tunnel syndrome (CTS) symptoms and their impact on functional abilities. Including this detailed summary aims to enhance transparency and facilitate reproducibility.

For our analysis, both the SSS and FSS scores were calculated as the **average** of the ratings across all items, rather than the total sum of the responses. This approach provides a standardized measure for easier interpretation and comparison.

Symptom Severity Scale (SSS)

Symptom Severity Scale (11 items)	1	2	3	4	5
1. How severe is the hand or wrist pain that you have at night?	Normal	Slight	Medium	Severe	Very serious
2. How often did hand or wrist pain wake you up during a typical night in the past two weeks?	Normal	Once	2 to 3 times	4 to 5 times	More than 5 times
3. Do you typically have pain in your hand or wrist during the daytime?	No pain	Slight	Medium	Severe	Very serious
4. How often do you have hand or wrist pain during daytime?	Normal	1-2 times / day	3-5 times / day	More than 5 times	Continued
5. How long on average does an episode of pain last during the daytime?	Normal	< 10 minutes	10 – 60 minutes	continued	> 60 minutes
6. Do you have numbness (loss of sensation) in your hand?	Normal	Slight	Medium	Severe	Very serious
7. Do you have weakness in your hand or wrist?	Normal	Slight	Medium	Severe	Very serious
8. Do you have tingling sensations in your hand?	Normal	Slight	Medium	Severe	Very serious
9. How severe is numbness (loss of	Normal	Slight	Medium	Severe	Very serious

sensation) or tingling at night?					
10. How often did hand numbness or tingling wake you up during a typical night during the past two weeks?	Normal	Once	2 to 3 times	To 5 times	More than 5 times
11. Do you have difficulty with the grasping and use of small objects such as keys or pens?	Without difficulty	Little difficulty	Moderate difficulty	Very difficult	Very difficult

Functional Status Scale (FSS)

The Functional Status Scale (FSS) consists of 8 questions that assess the impact of carpal tunnel syndrome on daily activities. Each activity is rated on a 5-point scale, with higher scores indicating greater difficulty (1 = No difficulty, 5 = Maximum difficulty).

Questions:
1. Writing.
2. Buttoning clothes.
3. Holding a book while reading.
4. Gripping a telephone handle.
5. Opening jars.
6. Household chores.
7. Carrying grocery bags.
8. Bathing and dressing.
